# A Hierarchical Spatial–Temporal Cross-Attention Scheme for Video Summarization Using Contrastive Learning

**DOI:** 10.3390/s22218275

**Published:** 2022-10-28

**Authors:** Xiaoyu Teng, Xiaolin Gui, Pan Xu, Jianglei Tong, Jian An, Yang Liu, Huilan Jiang

**Affiliations:** 1 Department of Faculty of Electronic and Information Engineering, Xi’an Jiaotong University, Xi’an 710049, China; 2 Shaanxi Province Key Laboratory of Computer Network, Xi’an Jiaotong University, Xi’an 710049, China; 3 Medical College, Northwest Minzu University, Lanzhou 730030, China; 4 ONYCOM Co., Ltd., Seoul 04519, Korea

**Keywords:** video summarization, spatial–temporal features, cross-attention

## Abstract

Video summarization (VS) is a widely used technique for facilitating the effective reading, fast comprehension, and effective retrieval of video content. Certain properties of the new video data, such as a lack of prominent emphasis and a fuzzy theme development border, disturb the original thinking mode based on video feature information. Moreover, it introduces new challenges to the extraction of video depth and breadth features. In addition, the diversity of user requirements creates additional complications for more accurate keyframe screening issues. To overcome these challenges, this paper proposes a hierarchical spatial–temporal cross-attention scheme for video summarization based on comparative learning. Graph attention networks (GAT) and the multi-head convolutional attention cell are used to extract local and depth features, while the GAT-adjusted bidirection ConvLSTM (DB-ConvLSTM) is used to extract global and breadth features. Furthermore, a spatial–temporal cross-attention-based ConvLSTM is developed for merging hierarchical characteristics and achieving more accurate screening in similar keyframes clusters. Verification experiments and comparative analysis demonstrate that our method outperforms state-of-the-art methods.

## 1. Introduction

With the rapid development of multimedia information technology and intelligent terminal equipment, video data have emerged as a critical medium of information transmission due to its lack of reading threshold and high data-carrying capacity. However, the openness and informality of video production result in the accelerated growth of video data and several undesirable phenomena, such as widespread data redundancy [1], unclear content emphasis, and blurred video theme boundaries. Therefore, it is becoming vital to provide effective and efficient tools for the management, browsing, and retrieval of these videos. Video summarization, which uses a subset of the most informative frames to create a condensed version of the original video by removing redundant information [2,3,4], is an effective tool for addressing these issues.

Recent methods for video summarization rely heavily on the superior performance of deep learning, particularly in feature extraction. In addition, feature extraction is a fundamental component of video summarization algorithms that extract time series [5,6] or spatial–temporal features from video data [7,8]. From the perspective of a video feature, the performance of the video summary is dependent on the feature extraction technique. These deep learning video summarization algorithms constantly increase the depth and breadth of video feature extraction to improve its performance. The most important criterion for measuring video summarization performance is user satisfaction. User satisfaction is contingent upon their requirements for video summarization performance. Furthermore, user requirements can be translated into property constraints of algorithms [7]. These property constraints can be categorized as representativeness [5], content coverage [8], redundancy [3], diversity [5], interestingness [9], importance [10], etc. The variety of user requirements continues to expand, while their feature definitions are more hazy. Consequently, video summarization algorithms focusing on video salient characteristics extraction are incapable of satisfying the multi-source user requirements. In addition, with the rise in popularity of video terminal equipment and the evolution of multimedia technology, hand-held and fragmented time-created videos have become the predominant sources of new created video data. Certain more prominent properties of the new video production, such as significant redundancy, a lack of strong focus, and a fuzzy theme boundary, disrupt the video summarization’s initial thinking mode based on video feature information and present it with new challenges. With the evolution of video characteristics and user requirements for video summarization, the demand for keyframe accuracy screening has increased. Some traditional methods are no longer applicable, such as clustering [11].

To be more precise, existing algorithms can meet a portion of the user-centered requirements and capture good summarization performance. However, the following challenges remain: contradiction between breadth extraction of salient video characteristics and multi-source of user diversified requirements; the contradiction between depth extraction of salient video characteristics and unbounded new video productions; the contradiction between similarity frames and more accurate keyframe screening.

**Figure 1:**
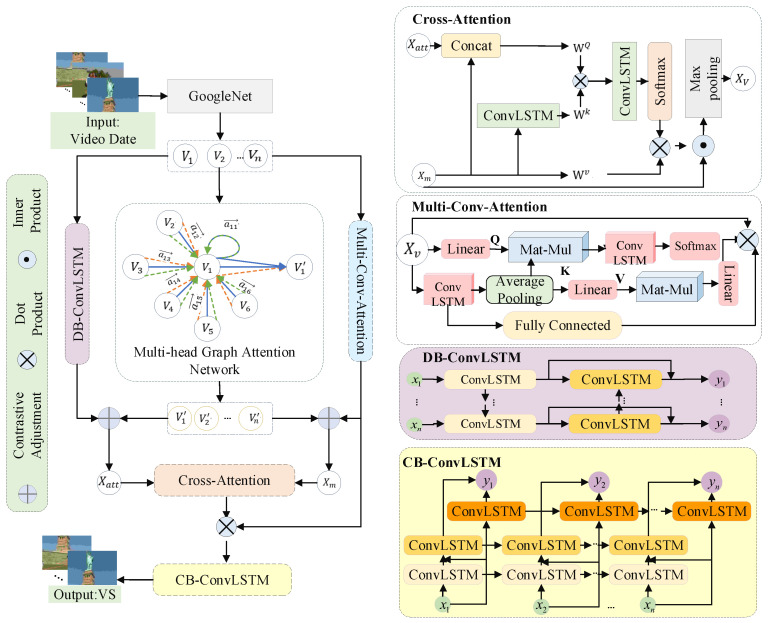
Overview of our approach.

To address the issues mentioned above, this paper proposes a hierarchical spatial–temporal cross-attention scheme based on contrastive learning, as shown in Figure 1. The central idea of this article is to extract features and relationships between frames that account for coarse and fine-grained, global and local, depth and breadth, to fuse hierarchical features while increasing the difference between similar frames, and then screen keyframes and generate summaries by evaluating their significance. From the perspective of video feature extraction, the solution to diverse user requirements for video summary lies in the extraction of the frame’s own characteristics, relationship features between frames, and relationship features between frames and the entire video. This study uses DB-ConvLSTM and multi-head attention mechanisms to design multi-conv-attention cells and joint GAT to acquire the spatial–temporal connection of keyframes to extract fine-grained spatial–temporal feature information from video frames. The GAT adjusted DB-ConvLSTM to extract the global and breadth features. In addition, to amplify the difference of similar keyframes, a spatial–temporal cross-attention-based ConvLSTM is constructed for merging hierarchical characteristics. Finally, video summarization is generated by CB-ConvLSTM through possibility. Therefore, the major contributions of this work can be summarized as follows:

1. A hierarchical spatial–temporal video feature extraction approach is developed. The purpose is to ensure as much characteristic information as possible for generating video summarization;
2. A cross-attention cell that combines the local and global features information based on DB-ConvLSTM is proposed. It seeks to emphasize the difference between related frames and achieve more accurate screening in similar keyframes clusters for video summary generation;
3. Verification experiments and comparative analysis are performed on two benchmark datasets (TVSum and SumMe) for this paper’s algorithm. The results demonstrate that the proposed algorithm is extremely rational, effective, and usable.

## 2. Related Work

In this section, we briefly overview some state-of-the-art video summarization approaches and correlation techniques pertaining to our hierarchical spatial–temporal cross-attention scheme.

### 2.1. Video Summarization

Generally speaking, pre-processing, feature extraction, post-processing, and VS creation comprise the video summary generating procedures. The post-processing can be left out. In particular, feature extraction is the central stage of the algorithm. The initial algorithm is based on time series techniques such as vsLSTM/dppLSTM [5]. The initial method of similar keyframes decision is based on clustering [11]. Zhao et al. [12] develop an extended bidirectional LSTM (Bi-LSTM) for extracting both structure and information characteristics from video data. To acquire a more precise extraction of video features, refs. [3,13] offer a keyframe-selection strategy based on video spatial–temporal characteristics. In addition, graph neural networks are employed to implement this notion [1,6]. However, the aforementioned algorithms are all video-centric and lack comprehensive analysis of video topics and user demands. In [14], first-person (egocentric) videos-based models are proposed. A model of characterizing egocentric video frames uses a graph-based center-surround model. User requirements impose certain restrictions on the feature extraction results. The video summarization algorithms [15] are based on attention technologies, mimicking human keyframe filtering. Ji et al. [16] solve the problem of short-term contextual attention insufficiency and distribution inconsistency. Köprü [17] proposes two new architectures based on temporal attention (TA-AVSUM) and spatial attention (SA-AVSUM).

Additionally, for the video summarization algorithm, both video feature information and video frame relational are crucial [18]. Continuously improving the performance of the user-requirements-driven algorithm fundamentally necessitates more comprehensive and accurate feature extraction. This scheme is based on the concept of creating stereoscopic modeling using spatial–temporal feature information, relationship information, and other multi-elements.

### 2.2. Cross Attention

Refs. [19,20,21,22] have conducted substantial study on how to more properly and completely extract video features and the relationship features between video frames. Contextual information is vital in visual understanding problems [19] and is also applicable to generating video summarization. Huang et al. [19] proposes a Criss-Cross Network (CCNet) based on attention for obtaining video information in a more effective and efficient way. Lin et al. [20] presents a universal Cross-Attention Transformer (CAT) module for accurate and efficient semantic similarity comparison in one-shot object detection. In [22], the attention mechanism is incorporated at two main levels: a self-attention module leverages global interactions between encoder features, while cross-attention in the skip connections allows fine spatial recovery in the U-Net decoder by filtering out non-semantic features. It can be seen that cross-attention has the ability to simultaneously extract the depth and breadth characteristics of video data. This study uses cross-attention to merge the hierarchical spatial–temporal characteristics, and it aims to accentuate the distinctions between video frames.

### 2.3. Graph Attention Networks (GATs)

Veličković et al. [23] give a novel neural network architecture that operates on graph-structured data, leveraging masked self-attentional layers to address the shortcomings of prior methods based on graph convolutions or their approximations. GATs provide distinct weights to each neighbor based on their importance, effectively filtering the neighbors. Zhong et al. [1] build a method for video summarizing utilizing graph attention networks and Bi-LSTM. However, it does not take into account information loss throughout the confrontation process. This paper makes use of GATs to capture spatial–temporal relational attention between video frames and comparative-adjusting feature extraction.

## 3. Materials and Methods

Figure 1 shows an overview of our hierarchical spatial–temporal cross-attention scheme for video summarization. DB-ConvLSTM, multi-conv-attention, and multi-head attention GAT are all used for video feature extraction. The DB-ConvLSTM is employed to extract coarse-grained global spatial–temporal video characteristics. Effective fine-grained local features are extracted using multi-conv-attention networks and spatial–temporal relational feature extraction using multi-head attention GAT. This research derives hierarchical spatial–temporal feature information on the basis of cross-attention, taking into consideration both global and local characteristics and coarse-grained and fine-grained features. In particular, this scheme promotes comparative learning for acquiring local feature information for multi-conv-attention and GAT, and obtaining global feature knowledge for DB-ConvLSTM and GAT. The local and global characteristics are combined using spatial–temporal cross-attention. Finally, CB-ConvLSTM obtains the video summary.

Following the algorithm phases, this part elaborates the DB-ConvLSTM and CB-ConvLSTM, contrastive adjustment learning, and spatial–temporal cross-attention for the keyframes screening module. The contrastive adjustment learning is adjustment learning based on contrastive learning. Finally, we will introduce the loss function used in our framework.

### 3.1. DB-ConvLSTM and CB-ConvLSTM

Both DB-ConvLSTM and CB-ConvLSTM are founded on the technology of ConvLSTM. ConvLSTM is not only designed for extracting spatial–temporal information features but also for inferring saliency information concurrently. Then, suppose there are *n* frames in a video, the whole video can be written as $f=\left\{f_{1},\cdots f_{n}\right\}$, $c_{t}$ is the memory cell, $f_{t}$ is the forget gate, and $i_{t}$ is the input gate. From [24], we can obtain that the ConvLSTM is defined as:

(1) $$\begin{array}{c} i_{t}=\sigma \left(W_{i}^{\chi }\times X_{{}_{t}}+W_{i}^{H}\times H_{t-1}\right)\\ f_{t}=\sigma \left(W_{f}^{\chi }\times X_{{}_{t}}+W_{f}^{H}\times H_{t-1}\right)\\ o_{t}=\sigma \left(W_{o}^{\chi }\times X_{{}_{t}}+W_{o}^{H}\times H_{t-1}\right)\\ c_{t}=f_{t}\circ c_{t-1}+i_{t}\circ \tanh\left(W_{c}^{\chi }\times X_{{}_{t}}+W_{c}^{H}\times H_{t-1}\right)\\ H_{t}=o_{t}\circ \tanh\left(c_{t}\right) \end{array}$$

#### 3.1.1. DB-ConvLSTM

In the video information processing methods, the DB-ConvLSTM [25] network is suggested to extract spatial–temporal video characteristics more deeply and precisely. DB-ConvLSTM is a bidirectional two-layer architecture, one forward-oriented and one backward-oriented. The forward-oriented and backward-oriented have information interaction. The deeper layer is composed of backward-cells, its input is the output features of forward-cells, and the output is ${\left\{Y_{t}\right\}}_{t=1}^{t}$. The backward-ConvLSTM is defined as:

(2) $$i_{t}^{b}=\sigma \left(W_{i}^{H^{f}}\times H_{{}_{t}}^{f}+W_{i}^{H^{b}}\times H_{t+1}^{b}\right)$$

(3) $$f_{t}^{b}=\sigma \left(W_{f}^{H^{f}}\times H_{{}_{t}}^{f}+W_{f}^{H^{b}}\times H_{t+1}^{b}\right)$$

(4) $$o_{t}^{b}=\sigma \left(W_{o}^{H^{f}}\times H_{{}_{t}}^{f}+W_{o}^{H^{b}}\times H_{t+1}^{b}\right)$$

(5) $$c_{t}^{b}={f_{t}}^{b}\circ c_{t+1}^{b}+i_{t}^{b}\circ \tanh\left(W_{c}^{H^{f}}\times H_{t}^{f}+W_{c}^{H^{b}}\times H_{t+1}^{b}\right)$$

(6) $$H_{t}^{b}=o_{t}^{b}\circ \tanh\left(c_{t}^{b}\right)$$

where *W* are the training parameters, denoting the learnable weights, *H* is the hidden state, σ is the activation function, × denotes the convolution operator, and ∘ denotes the hadamard product. In the VS algorithm, the DB-ConvLSTM can be written as:

(7) $$Y_{t}=\tanh\left(W_{y}^{H^{f}}\times H_{t}^{f}+W_{y}^{H^{b}}\times H_{t-1}^{b}\right)$$

tanh is the activation function to normalize $Y_{t}$, and the loss function of training DB-ConvLSTM is distance minimization.

#### 3.1.2. CB-ConvLSTM

CB-ConvLSTM is capable of extracting not only the characteristics of a single video frame but also the spatial–temporal relationships between different frames [7]. From [7], we can obtain the definition of CB-ConvLSTM, based on Equations (2)–(7), and replace the content in Equation (1) by ConvLSTM; then, CB-ConvLSTM is defined as follows:

(8) $$\begin{array}{c} H_{t}^{f}=ConvLSTM\left(X_{t},H_{t-1}^{f}\right) \end{array}$$

(9) $$\begin{array}{c} H_{t}^{b}=ConvLSTM\left(X_{t}⊕H_{1,t},H_{t+1}^{b}\right) \end{array}$$

⊕ is the operation of fusing two vectors, $H_{1,t}$ is the first hidden state, and the loss function of training CB-ConvLSTM is distance minimization. In this paper, the three layers in the network cell aim to extract and aggregate the features, and the final outputs are the possibility of whether a frame will be selected as a keyframe for video summarization.

### 3.2. Contrastive Adjustment Learning

**Figure 2:**
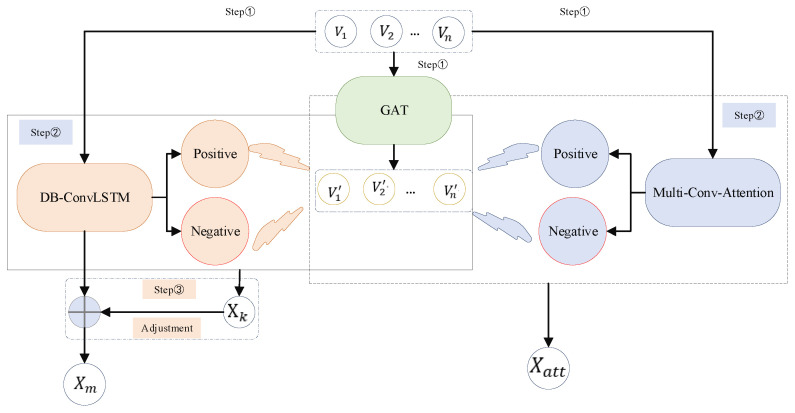
Step of contrastive adjustment learning.

Contrastive learning [26] introduces a novel idea of features derived from many perspectives: the learning algorithm does not have to concentrate on every element of the sample itself, as long as it learns enough traits to differentiate it from others. In our study, the use of contrastive learning serves three purposes: (1) to overcome the diversity theme of video, (2) to extract elastic traffic feature information, and (3) to increase feature extraction with surface breadth and detail while enlarging the difference between video frames. As shown in Figure 2, the specific application of our strategy is to use the GATs-obtained data as the primary line and generate positive and negative pairs from the results of DB-ConvLSTM and multi-conv-attention, respectively. $D_{m}$ is supposed as the results of the two sections of the comparative learning. DDPG [27] is used to train the $D_{m}$ adjusted DB-ConvLSTM, which is the same as [1].

$x^{+}$ is the positive sample, and $x^{-}$ is the negative sample, *S* is the function for measuring the samples’ similarity, and similar to [26], the rule for setting positive pairs is:

(10) $$S\left(Y\left(x\right),Y\left(x^{+}\right)\right)\gg S\left(Y\left(x\right),Y\left(x^{-}\right)\right)$$

$D_{t}$ is the video characteristics, which are extracted by multi-conv-attention and DB-ConvLSTM. $D^{+}$ is the keyframe sets, $D^{-}$ is the non-keyframe sets, $Q_{j}$ is feature mapping of the labeled data, and $Q^{+}$ is the annotated manually keyframe-sets. Then, the positive pairs include: $Y^{A}=\left\{D^{+}\left(x\right)\cap Q^{+}\left(x\right)\right\}$. Moreover, the loss function of a negative sample is InfoNCE in this paper, and it can be written as:

(11) $$L_{adj}=\sum_{x,x^{+},x^{-}}\left[-log\left(\frac{e^{Y{\left(x\right)}^{T}Y\left(x^{+}\right)}}{e^{Y{\left(x\right)}^{T}Y\left(x^{+}\right)}{+}^{Y{\left(x\right)}^{T}Y\left(x^{-}\right)}}\right)\right]$$

### 3.3. Multi-Conv-Attention and Cross-Attention

#### 3.3.1. Multi-Conv-Attention

The temporal, spatial, and multi-element video properties are all important parts of our approach. As a consequence, a new network cell is constructed using ConvLSTM and multi-head attention. It uses convolution to improve the attention mechanism’s ability to get as much video information as possible. In our multi-conv-attention cell, we first adopt a set of projections to obtain query *Q*. Additionally, it employs ConvLSTM and average pooling to produce two sets of projections of key *K* and value *V*, enhancing the *K* and *V* dimensions of the attention mechanism while also boosting the performance and consistency of feature information extraction. Finally, the attention is calculated as:

(12) $$\begin{array}{c} M_{c}\left(Q,K,V\right)=Softmax\left(ConvLSTM\left(\frac{QK^{T}}{\sqrt{d_{k}}}\right)\right)V \end{array}$$

In this scheme, we employ n=8, and $d_{k}=d_{v}=d_{model}/n=64$.

#### 3.3.2. Cross-Attention

**Figure 3:**
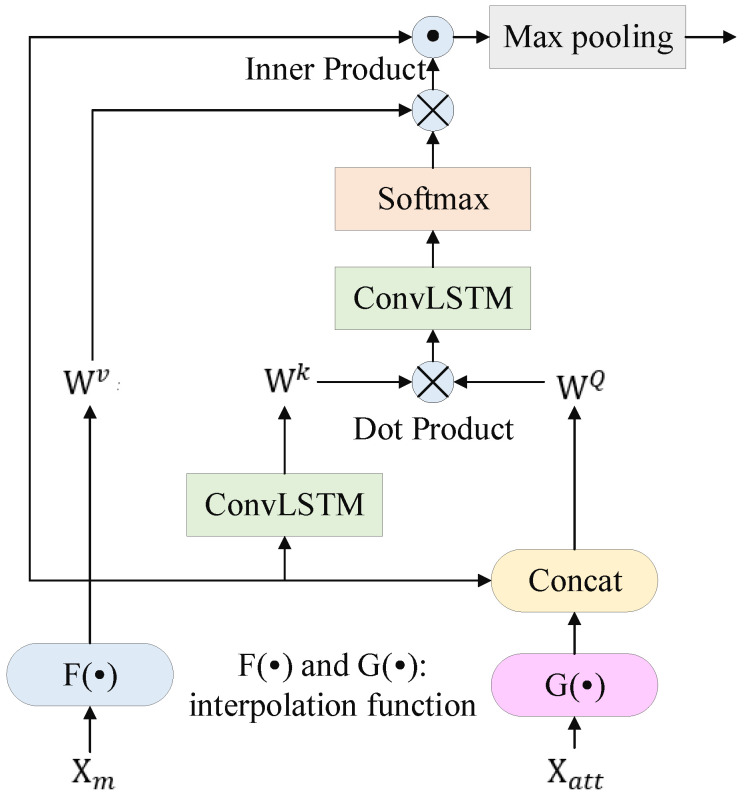
Spatial–temporal cross-attention cell.

The Cross-Attention module is shown in Figure 3. $F\left(\cdot \right)$ and $G\left(\cdot \right)$ are projections to align dimensions using interpolation function. Then, the module performs cross-attention between $X_{m}$ and $X_{att}$, which can be expressed as

(13) $$q=G\left(X_{m}\right)\cdot W^{Q}$$

(14) $$k=ConvLSTM\left(G\left(X_{att}\right)\right)\cdot W^{k}$$

(15) $$v=F\left(X_{m}\right)\cdot W^{v}$$

Finally, calculate the cross-attention using Equation (12).

### 3.4. Loss Function

The total loss is primarily made up of three components, and all the loss functions of these parts are based on cross-entropy. In items of supervised learning, the selection of keyframes is ultimately intended to decrease the discrepancy between predicted and background data. The cross-entropy is used to approximate the distribution of the learnt model to the background data. The lower the value is, the more similar the probability distributions of the anticipated and background data. *p* is the probability distribution of background data, and *q* is the predicted probability distribution, and the cross-entropy $H\left(p,q\right)$ is:

(16) $$H\left(p,q\right)=\sum_{i=1}^{n}p_{i}log\frac{1}{q_{i}}=-\sum_{i=1}^{n}p_{i}logq_{i}$$

In our network, the softmax is used to normalize the cross-entropy, $y_{i}$ is the output of network cells, ${\hat{y}}_{i}$ is the category *i* of background data, ${\hat{y}}_{i}\in \left\{0,1\right\}$, and the loss function is:

(17) $$L=-\frac{1}{m}\left[\sum_{i=1}^{m}{\hat{y}}_{i}log\frac{e^{z_{i}}}{\sum_{i=1}^{k}e^{z_{k}}}\right]=-\frac{1}{m}\left[\sum_{m}^{i=1}{\hat{y}}_{i}logy_{i}\right]$$

$L_{mat}$ is the loss function of the model of multi-conv-attention contrastive GAT. $L_{dat}$ is the loss function of the model of DB-ConvLSTM contrastive adjustment GAT. $L_{cro}$ is the loss function of cross-attention. Both $L_{mat}$ and $L_{dat}$ are cross-entropy, as defined by Equation (17). To resolve the centralization issue and reduce the ambiguity problem in key frame filtering, we use $L_{cen}$ for centralization keyframe scores:

(18) $$L_{cen}=\lambda \cdot \frac{min(L_{dat},L_{mat})}{max(L_{dat},L_{mat})}$$

In Equation (17), λ balances the function of global and local domains. Formally, the objective function $L_{obj}$ is written as

(19) $$L_{tol}=\mu \cdot L_{cro}+L_{cen}$$

μ balances the loss of cross-attention and multi-conv-attention.

## 4. Experiments Analysis

### 4.1. Datasets

Each database has its focus, so before the experiment, the two databases TVsum [28] and SumMe [29] should be analyzed, and the results are shown in Table 1. Additionally, we use two other public datasets, OVP (Open Video Project) [30] and YouTube [11], to augment the training sets.

### 4.2. Evaluation Metrics

To facilitate a comparison study of the experimental influence on current research findings, the Precision, Recall, and F-score are used as measurement standards, similar to the literature [3]. *S* is the video summarization generated by the algorithm, *G* denotes the ground user-marked ground truth, and the following definitions apply to Precision, Recall, and F-score:

(20) $$Precision=\frac{\left|S\cup G\right|}{\left|S\right|}$$

(21) $$Recall=\frac{\left|S\cup G\right|}{\left|G\right|}$$

(22) $$F-score=\frac{2\times Precision\times Recall}{Precision+Recall}$$

As shown in [31], randomly generated video summaries may achieve equivalent performance when using the F-score measure. To avoid this problem, we evaluated our method as seen in Table 2. Under our comparison method, the comparing parameter is F-score. Furthermore, to be more precise, these datasets are randomly split into different training and testing sets five times, and the final measure is produced by averaging the five results.

### 4.3. Experimental Environment and Parameters Settings

**Figure 4:**
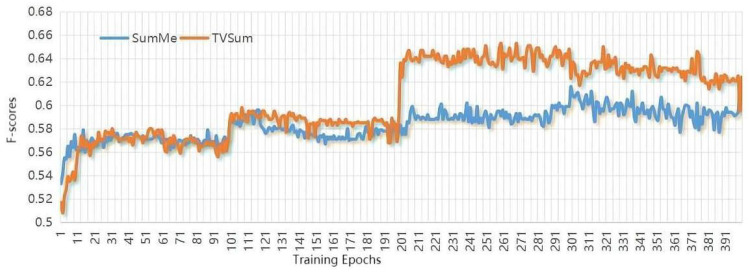
Plots show the influence of training epochs on the value of F-scores.

**Figure 5:**
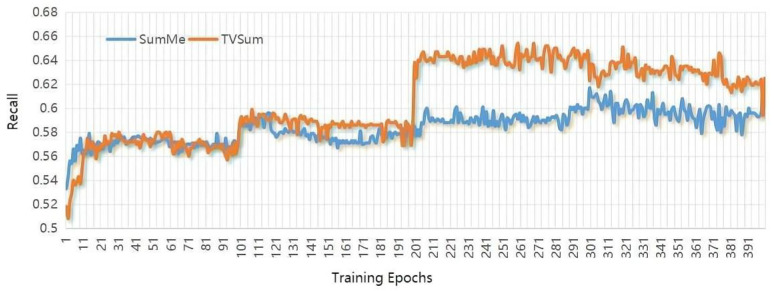
Plots show the influence of training epochs on the value of Recall.

**Figure 6:**
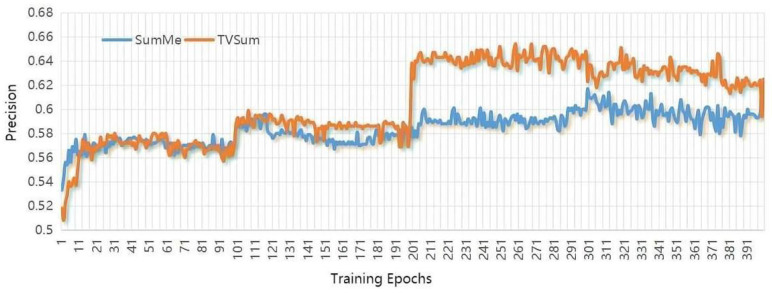
Plots show the influence of training epochs on the value of Precision.

The deep learning platform for operating our approach is Pytorch. The hidden states are with dimensionality of 256 for ConvLSTM, and other parameters settings are as follows: similar to other algorithms, we use the pool5 layer of GoogleNet to extract the visual features for each video frame. The number of ConvLSTMs hidden layers is 256, the learning rate initialized is le−5, the batch size is 5, the kernel size is set as (5,1), and the maximum training epoch is set as 100. Furthermore, considering that the training epochs are critical to summarization performance, after increasing for five epochs continuously, their influence on the validation set is plotted in Figure 4, Figure 5 and Figure 6. The horizontal coordinate is the training epochs, and the vertical coordinates are the values the of F-scores, Recall, and Precision.

### 4.4. Comparative Analysis of Schemes

This section verifies the feasibility and effectiveness of the proposed strategy through two ways: one is validation of the algorithm itself, and the other one is comparative analysis with state-of-the-art video summarization approaches.

#### 4.4.1. Self-Verification

**Figure 7:**
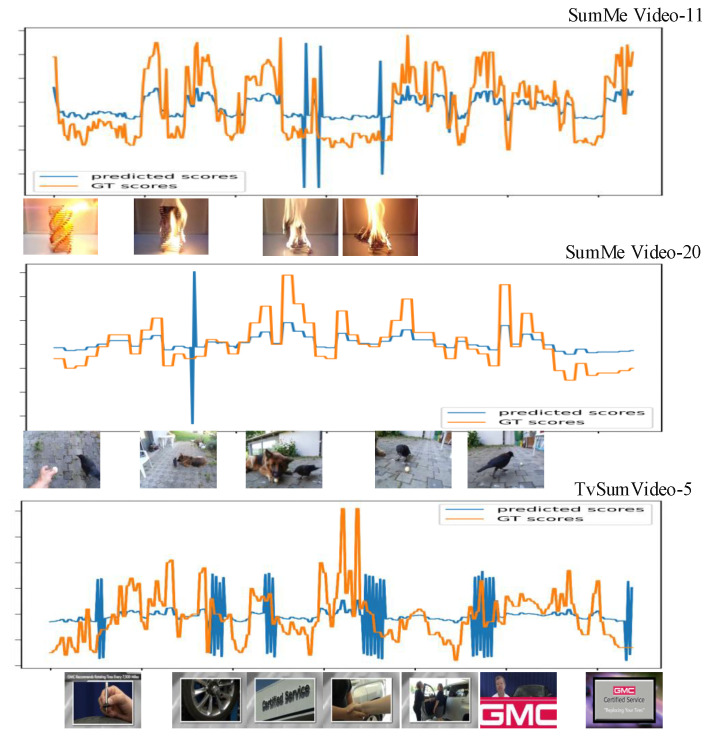
An example of generating video summarization on SumMe and TVSum datasets; the first two are samples from SumMe datasets and the last one is from TVSum datasets. The yellow lines show the annotation importance scores of ground truth summarization marked by the user, and the blue lines show the prediction score of our method.

**Figure 8:**
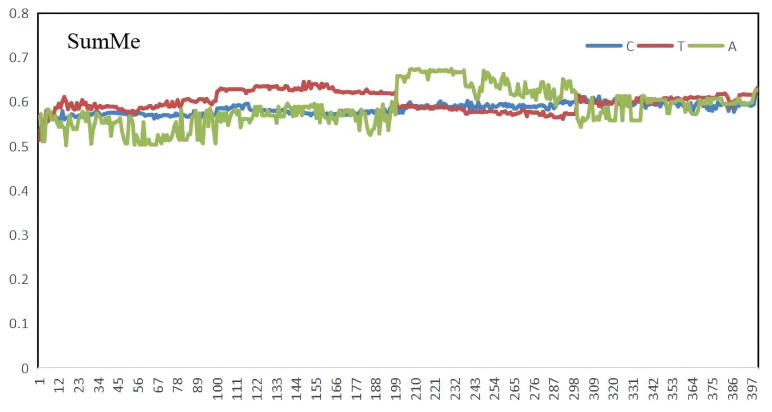
The A, C, and T results of SumMe.

**Figure 9:**
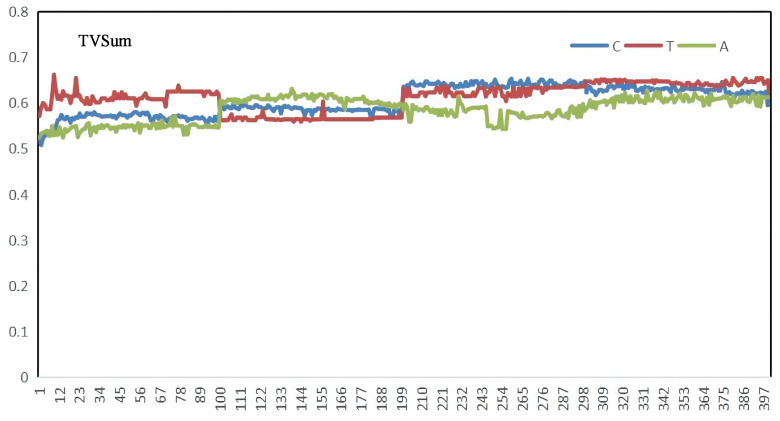
The A, C, and T results of TvSum.

Before comparing the scheme to other state-of-the-art algorithms, it is vital to validate the scheme’s performance itself. Table 3 and Figure 7 show the results of evaluating the performances of our methods on the SumMe and TVSum datasets. From the perspective of result stability, the test variation curves of C, T, and A on SumMe and TVSum datasets are shown in Figure 8 and Figure 9. The horizontal coordinate of both figures is training epochs. Figure 7 gives an example of generating video summarization on the SumMe and TVSum datasets by our approach; the yellow lines show the annotation importance scores of ground truth summarization marked by the user, and the blue lines show the prediction score of our method. We clearly observe that our models achieve very competitive results against state-of-the-art methods.

#### 4.4.2. Comparative Analysis with Relative Approaches

The primary components of our algorithm consist of the attention mechanism, ConvLSTM, and GATs. In this section, we compared our approach with some state-of-the-art video summarization methods on SumMe and TvSum. Comparison methods can be classified into three categories: based on “LSTM+”, based on “Attention+”, and based on GATs methods.

(1) Comparison With “Bi-LSTM+” Methods

Due to the few research results on the summary algorithm based on ConvLSTM, this section compares our scheme to the Bi-LSTM based algorithms. Some classic algorithms are compared, as shown in Table 4.

H-RNN [12] and HAS-RNN [32] are based on hierarchical architecture. According to the findings of the comparison, we observe that our method outperforms state-of-the-art video summarization methods on both datasets.

(2) Comparison With “Attention+” Methods

Since the scheme in this paper involves not only the combination of ConvLSTM and attention but also the graph neural network, we will analyze it separately. The results of comparison with “Attention+” methods are shown in Table 5. SABTNet [15] is based on attention and a binary neural tree. Liang et al. [34] proposes a video summarization method based on dual-path attention, while Zhu et al. [35] is based on hierarchical attention. Table 5 demonstrates that the cross-attention method has clear benefits over the SumMe database.

(3) Comparison With “Graph Attention+” Methods

The extraction of spatial–temporal characteristics and frame–relationship features is facilitated by a graph neural network. Table 6 shows the results of comparing our method with some “Graph Attention+” video summarization methods including RSGN [13], GCAN [37], Bi-GAT [1] and SumGraph [38]. From the experimental results in Table 6, our method outperforms other approaches, which are based on “Graph Attention+”.

#### 4.4.3. Comparison Results

Following the comparison tests outlined above, it can be seen that the proposed method has certain advantages over existing approaches, most notably in the SumMe database set. Specifically, the hierarchical spatial–temporal cross-attention scheme in this research enhances the algorithm’s stability, scalability, and other performance characteristics.

## 5. Conclusions

This paper proposes a hierarchical spatial–temporal cross-attention scheme for video summarization using contrastive learning. The scheme solves the contradictions of diversification user requirements, depth and breadth of features extraction and new creation videos. The hierarchical architecture is divided primarily into depth and breadth feature extraction and spatial–temporal cross-attention feature merging. This paper extracts local and depth features using a graph attention network and multi-head attention mechanism, and it extracts global and breadth features using a GAT adjusted DB-ConvLSTM. Furthermore, merging hierarchical characteristics via spatial–temporal cross-attention cells is used for more precise keyframe screening. Finally, video summarization is generated by CB-ConvLSTM. In practice, results from the TVSum and SumMe datasets indicate that the proposed algorithm is highly rational, effective, and usable. Nevertheless, the analysis of similarity keyframe screening is still insufficiently detailed.

## Figures and Tables

**Table 1:** Analysis of TVsum and SumMe dataset.

Datasets	Description
TVsum	The title-based video summarization dataset contains 50 videos of various genres (e.g., news, documentary, egocentric) and 1000 annotations of shot-level importance scores (20 user annotations per video). The duration varies from 2 to 10 min.
SumMe	The SumMe dataset consists of 25 videos, each annotated with at least 15 human annotated summaries. The duration of videos varies from 1.5 to 6.5 min.

**Table 2:** Datasets setting used for evaluation (C: Canonical; A: Augmented; T: Transfer).

Datasets	Setting	Training Phase	Testing Phase
TVSum	C	80% TVSum	The rest 20% of TVSum
	A	80% TVSum+SumMe+	The rest 20% of TVSum
		OVP+YouTube	
	T	SumMe+OVP+YouTube	TVSum
SumMe	C	80% SumMe	The rest 20% of SumMe
	A	TVSum+80% SumMe+	The rest 20% of SumMe
		OVP+YouTube	
	T	TVSum+OVP+YouTube	SumMe

**Table 3:** Performance analysis of self-verification (F-scores).

Data Sets	TVSum			SumMe		
**Metric**	**C (%)**	**A (%)**	**T (%)**	**C (%)**	**A (%)**	**T (%)**
MAX	65.3	67.4	66.2	61.6	63.1	64.5
MIN	50.8	50.2	55.9	53.3	52.4	51.3
AVERAGE	60.57	58.62	61.26	58.4	58.4	60.01

**Table 4:** Performance analysis of methods based on “Bi-LSTM+”.

Data Sets	TVSum			SumMe		
**Metric**	**C (%)**	**A (%)**	**T (%)**	**C (%)**	**A (%)**	**T (%)**
vsLSTM [5]	54.2	57.9	56.9	37.6	41.6	40.7
dppLSTM [5]	54.7	59.6	58.7	38.6	42.9	41.8
H-RNN [12]	57.9	61.9	−	42.1	43.8	−
HAS-RNN [32]	58.7	59.8	−	42.3	42.1	−
DHAVS [33]	60.8	61.2	57.5	45.6	46.5	43.5
Ours	65.3	67.4	66.2	58.4	58.4	60.01

**Table 5:** Performance analysis of methods based on “Attention+”.

Data Sets	TVSum			SumMe		
**Metric**	**C (%)**	**A (%)**	**T (%)**	**C (%)**	**A (%)**	**T (%)**
M-AVS [36]	61.0	61.8	−	44.4	41.6	−
SABTNet [15]	61.0	−	−	51.7	−	−
[34]	61.58	61.2	58.9	51.7	52.1	44.1
[35]	61.5	62.8	56.7	51.1	52.1	45.6
Interp-SUM [2]	59.14	−	−	47.7	−	−
3DST-UNet [3]	58.3	58.9	56.1	47.4	49.9	47.9
Ours	65.3	67.4	66.2	58.4	58.4	60.01

**Table 6:** Performance analysis of methods based on “Graph Attention+”.

Data Sets	TvSum (F-Score %)	SumMe (F-Score %)
RSGN [13]	60.1	45.0
GCAN [37]	60.1	53.0
Bi-GAT [1]	59.6	51.7
SumGraph [38]	63.9	51.4
Ours	65.36	58.48

## Data Availability

Not applicable.
